# Analysis of transmitted HIV-1 drug resistance using 454 ultra-deep-sequencing and the DeepChek^®^-HIV system

**DOI:** 10.7448/IAS.17.4.19752

**Published:** 2014-11-02

**Authors:** Ana Garcia-Diaz, Adele McCormick, Clare Booth, Dimitri Gonzalez, Chalom Sayada, Tanzina Haque, Margaret Johnson, Daniel Webster

**Affiliations:** 1 Virology, Royal Free London NHS Foundation Trust, London, UK; 2 R&D, ABL TherapyEdge Spain SL, Barcelona, Spain; 3 R&D, ABL SA, Luxembourg, Luxembourg

## Abstract

**Introduction:**

Next-generation sequencing (NGS) is capable of detecting resistance-associated mutations (RAMs) present at frequencies of 1% or below. Several studies have found that baseline low-frequency RAMs are associated with failure to first-line HAART. One major limitation to the expansion of this technology in routine diagnostics is the complexity and laboriousness integral to bioinformatics analysis. DeepChek (ABL, TherapyEdge) is a CE-marked software that allows automated analysis and resistance interpretation of NGS data.

**Objective:**

To evaluate the use of 454 ultra-deep-sequencing (Roche^®^ 454, Life Sciences; 454-UDS) and DeepChek for routine baseline resistance testing in a clinical diagnostic laboratory.

**Methods:**

107 newly diagnosed HIV-1-infected patients (subtypes: A, n=9; B, n=52; C, n=21; D, n=2; F, n=3; G, n=1; CRF01, n=7; CRF02, n=7; CRF06, n=1; CRF07, n=1; CRF10, n=1 and unassigned complex, n=2) with a median plasma viral load of 88,727 copies/mL (range: 1380–2,143,543) were tested by 454-UDS and Sanger sequencing for the detection of protease and reverse transcriptase RAMs. In addition, integrase RAMs were investigated in 57 of them. Sequence analysis and resistance interpretation were performed using DeepChek applying 1% and 20% thresholds for variant detections; filters applied were comparison between Sanger and 454-UDS, and Stanford and IAS list for resistance interpretation.

**Results:**

The time elapsed from generation of raw 454 data (between 2,000–5,000 sequences/sample) to elaboration of a resistance report was approximately 10 minutes per sample, equivalent to the time required for the same process using Sanger sequencing. Four patients (3.7%) showed baseline resistance by Sanger and 454-UDS at frequencies above 20%, which affected both NRTIs (n=2) and NNRTIs (n=2). In addition, 12 patients (11.2%) showed transmitted drug resistance (TDR) by 454-UDS at frequencies below 20% affecting NRTIs (n=9), NNRTIs (n=7) and PIs (n=2). Integrase resistance was not detected at baseline by 454-UDS or Sanger sequencing.

**Conclusions:**

DeepChek allowed easy and rapid analysis and interpretation of NGS data, thus facilitating the incorporation of this technology in routine diagnostics. The use of NGS considerably increased the detection rates of TDR to NRTI, NNRTIs and PIs. No transmitted resistance to integrase inhibitors was found in our population by Sanger sequencing or UDS.

